# The Influence of Ball Burnishing on Friction in Lubricated Sliding

**DOI:** 10.3390/ma13215027

**Published:** 2020-11-07

**Authors:** Slawomir Swirad, Pawel Pawlus

**Affiliations:** Faculty of Mechanical Engineering and Aeronautics, Rzeszow University of Technology, Powstancow Warszawy 8 Street, 35-959 Rzeszow, Poland; ppawlus@prz.edu.pl

**Keywords:** ball burnishing, coefficient of friction, surface topography

## Abstract

Ball burnishing treatment, using the Ecoroll system, of steel samples was conducted. In the experiment, the burnishing pressure was changed. After the treatments, measurements of the surface topographies of disc samples were conducted using a white light interferometer Talysurf CCI Lite. Tribological tests were carried out in a ball-on-disc configuration. After these tests, measurements of surface topographies of disc samples were repeated. Worn surfaces were also analyzed with a scanning electron microscope. It was found that as the result of burnishing, surface topography height of tested samples decreased. Ball burnishing led to a reduction in the frictional resistance. The highest friction reduction of more than 40% was achieved with a burnishing pressure of 20 MPa.

## 1. Introduction

Ball burnishing is a loss-free finishing process used for improving surface quality. In addition, compressive residual stresses are introduced with an increase in hardness, leading to an improvement in the subsurface integrity [1,2]. In this process, a hard ball is pressed against the sample and slides over its surface [3]. Surface and subsurface integrity can be improved by plastic deformation. Ball burnishing can be combined with other treatments, such as turning [1], grinding [4] and superfinishing [5]. Researchers typically obtained a reduction in surface height as a result of burnishing [6,7,8,9,10,11,12]. An increase in microhardness was reported in [11,12,13,14], while the addition of compressive residual stresses was reported in [1,12,14]. Various burnishing parameters are related to a reduction in surface roughness due to burnishing. For instance, Korzynski and Zarski [7] changed the burnishing force and the tool diameter, Skoczylas and Swirad [15] changed the pressure and the burnishing rate and Dzionk et al. [16] changed feed and burnishing speed.

The improvement of functional properties of samples after ball burnishing is typically attributed to subsurface properties such as microhardness and residual stresses [17,18,19].

However, surface topography of burnished samples can also affect frictional resistance, especially in lubricated conditions. It is difficult to find technical literature papers presenting the effects of ball burnishing on the frictional behaviors of sliding pairs—only results related to surfaces after other treatments have been presented. Dzierwa et al. [20] found that smoother surfaces produced less friction in pin-on-disc lubricated contact. The friction force was proportional to roughness height in studies conducted by Sedlacek et al. [21,22]. The friction force between nanometer thick lubricant films was higher when the composite rms. (root mean square) height of two solid surfaces was also higher [23]. However, the opposed effects of surface roughness height on lubricated friction were also obtained. In the work [24], the friction of rough steel surfaces was higher compared to that of smooth surfaces under boundary lubrication.

The height of surface texture is typically assessed by averaged parameters such as the standard deviation of height (Sq) [25]. However, for two-process textures, the standard deviation of the plateau height affected the friction coefficient. Two-process surfaces have tracks of two-machining processes. They were prepared to resemble textures created during running-in. They combine good sliding properties of smooth surfaces with the ability to maintain the oil of porous textures. Jeng [26] found that in mixed lubrication, two-process surfaces yielded less friction that a one-process texture, of similar standard deviation of height. Cylinder texture after finish honing and plateau honing was one of the earlier examples of two-process surfaces [27,28,29]. Plateau honing is also one of the first examples of surface texturing, which depends on the creation of connected or isolated dimples on sliding surfaces. The dimple can be a micro-hydrodynamic bearing in full or mixed lubrication, a micro-reservoir for lubricant in starved lubrication or a micro-trap for wear debris [30,31,32].

As aforementioned, it is difficult to find in technical literature publications about the impact of the ball burnishing process on the frictional resistance in lubricated contact. The present authors of this study try to fill this gap.

## 2. Experimental Details

Ball burnishing treatment, using the Ecoroll system, of samples from 42CrMo4 (chromium-molybdenum steel for quenching and tempering) steel of 34 ± 2 HRC hardness, was carried out using the CNC Mill Center VF-1 (Haas Automation Inc., Oxnard, CA, USA). In the experiment, the burnishing pressure, delivered by a hydraulic pump, was the input parameter. There were the following values of the burnishing pressure (P): 10, 20, 30 and 40 MPa. Burnishing speed was 0.5 m/min, while burnishing width was 0.01 mm. The samples were milled before the burnishing process. Table 1 presents the parameters of milling.

After the treatments, the surface topographies of milled and burnished samples were measured using an optical profilometer (Talysurf CCI Lite (Taylor Hobson Ltd., Leicester, UK)) of 0.01 nm vertical resolution. The objective 5× was used, so the measuring areas (containing 1024 × 1024 points) were 3.29 mm × 3.29 mm. Parameters of disc surface texture were computed using TalyMap 6 software (Digital Surf, Besancon, France). The form was eliminated by surface leveling. Surface topographies of analyzed samples were also measured after tribological tests and analyzed using SEM. SEM images were acquired with the Phenom ProX desktop scanning electron microscope equipped with a thermionic CeB_6_ (cerium hexaboride) source and a high sensitivity multi-mode backscatter detector (BSD). The SEM measurement was performed at a voltage of 10 kV, the imaging magnifications were commonly fixed 620 times. The sizes of the images of the SEM were 1024 × 1024. The actual scales per pixel were commonly set to 0.423 μm × 0.423 μm.

Tribological tests were conducted in a sphere-on-flat configuration under starved lubricated conditions. Before each test, 0.5 mL of L-AN-46 oil was supplied to the inlet side of the contact zone. This lubricant, used in earlier research [31,32], has the following properties: kinematic viscosity at 40 °C: 46.0 mm^2^/s, kinematic viscosity at 100 °C: 6.66 mm^2^/s, viscosity index: 96, ignition temperature: minimum 170 °C, flow temperature: maximum −12 °C and density at 15 °C: 880 kg/m^3^.

Steel discs co-acted with the ball made from 100Cr6 steel of 60 HRC hardness. The friction radius was 5 mm. The friction force was measured during tests. Tests were carried out at ambient temperature. The number of test repetitions was 3. Table 2 presents tribological parameters.

**Figure 1:**
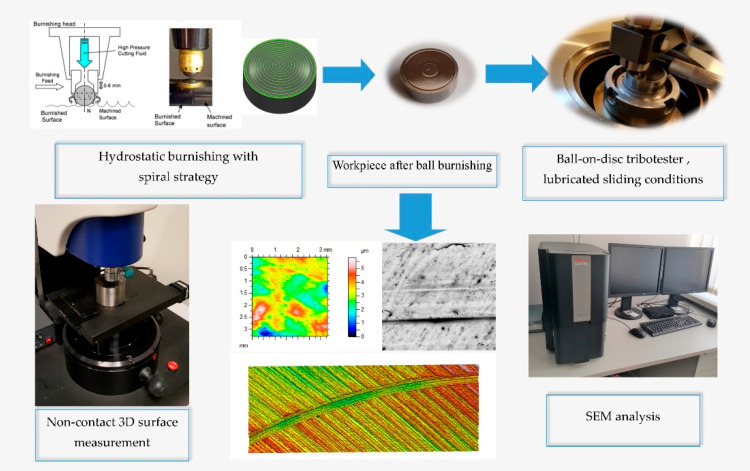
Scheme of experiments.

Dimensions of samples were determined by construction of a tribological tester (the diameter was 25.4 mm, the height was 9 mm). Figure 1 presents the scheme of experiments.

## 3. Surface Texture Analysis of Disc Samples before Tribological Tests

**Figure 2:**
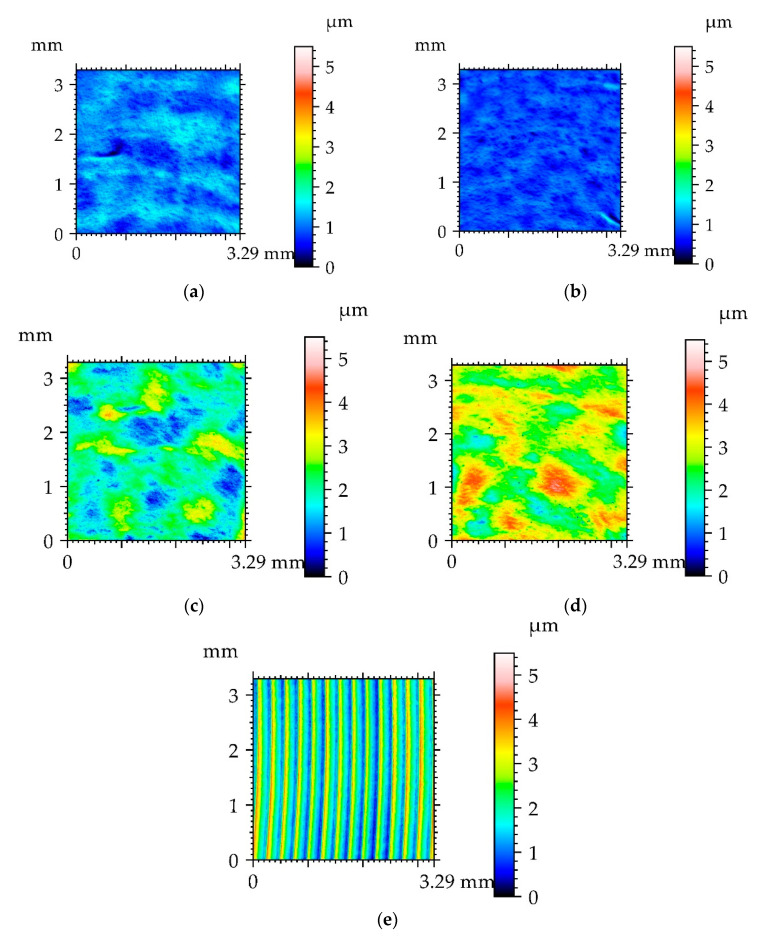
Contour plots of disc surfaces after burnishing with a pressure (P) of 10 MPa (**a**), 20 MPa (**b**), 30 MPa (**c**), 40 MPa (**d**) and after milling (**e**).

**Figure 3:**
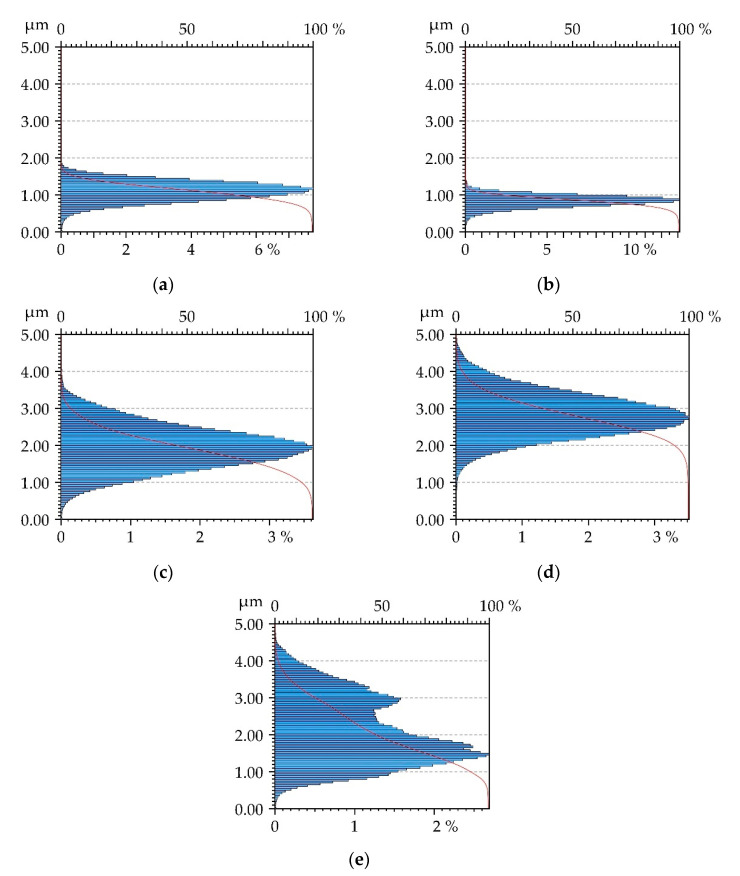
Material ratio curves and ordinate distributions of disc surfaces after burnishing with a pressure (P) of 10 MPa (**a**), 20 MPa (**b**), 30 MPa (**c**), 40 MPa (**d**) and after milling (**e**).

**Figure 4:**
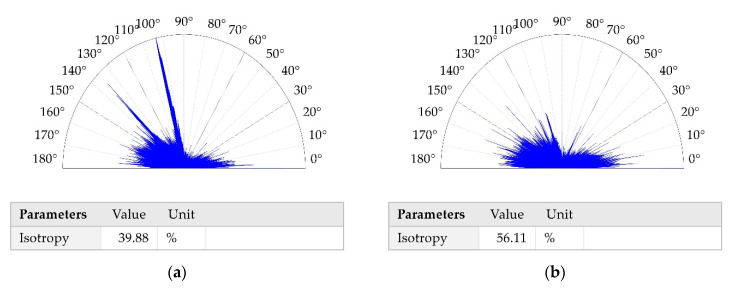

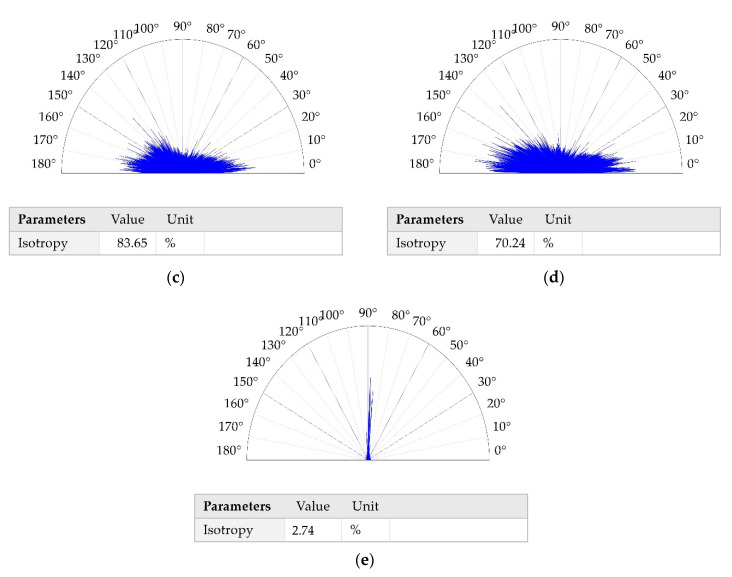
Directionality plots of disc surfaces after burnishing with a pressure (P) of 10 MPa (**a**), 20 MPa (**b**), 30 MPa (**c**), 40 MPa (**d**) and after milling (**e**).

**Figure 5:**
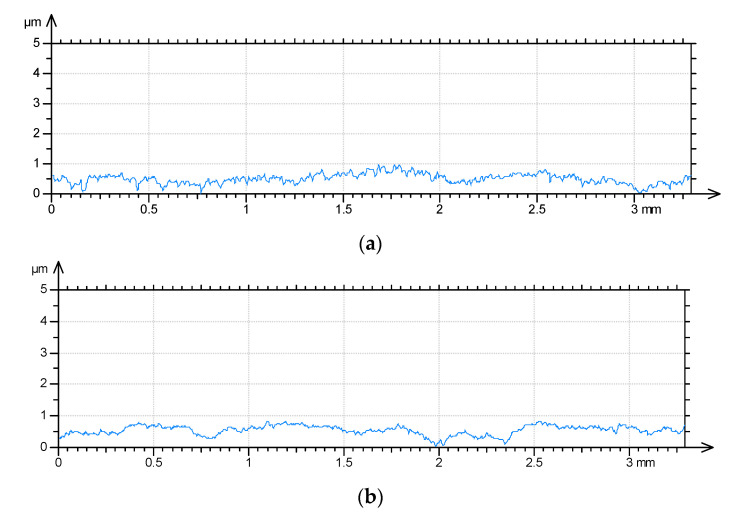

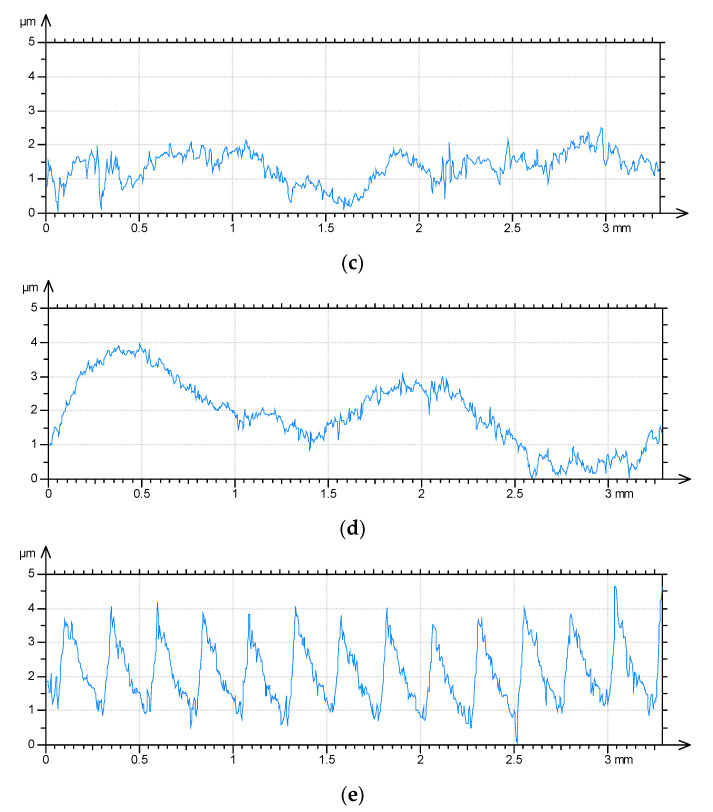
Representative profiles of burnished disc surfaces when pressure (P) was 10 MPa (**a**), 20 MPa (**b**), 30 MPa (**c**), 40 MPa (**d**) and of a milled disc surface (**e**).

Table 3 presents the results of surface texture analysis after the treatments. The following parameters from ISO 25178 (Geometric Product Specifications (GPS)—Surface texture: areal) standard were selected: root mean square height (Sq), skewness (Ssk), kurtosis (Sku), maximum peak height (Sp), maximum valley depth (Sv), auto-correlation length (Sal), texture–aspect ratio (Str), root mean square slope (Sdq), density of peaks (Spd) and arithmetic mean peak curvature (Spc). Book [25] presents definitions of these parameters. Figure 2 shows contour plots, Figure 3 presents material ratio curves and ordinate distributions, while Figure 4 shows directionality plots of the analyzed disc samples. Representative profiles of disc samples are presented in Figure 5.

One can see from the analysis of Table 3 and Figure 2, Figure 3 and Figure 5 that the surface after milling is the roughest, followed by surfaces after burnishing. A reduction in surface height was obtained as a result of the burnishing treatment. Burnishing also led to increases in the spatial parameters Sal and Str and to decreases in parameters Sdq and Spc. The smallest height characterized by the Sq, Sp and Sv parameters was achieved for the pressure (P) of 20 MPa, followed by the lowest burnishing pressure and higher pressures of 30 and 40 MPa. When the material is above the mean plane, the surface is negatively skewed, and in the opposite case, the surface is positively skewed [33,34]. The values of skewness (Ssk) of burnished textures were similar to 0; for lower burnishing pressures of 10 and 20 MPa, they were a little negative, while for higher pressures of 30 and 40 MPa, they were marginally positive. However, the ordinate distributions of burnished surfaces were symmetrical. The kurtosis (Sku) defines how heavily the tails of texture height distribution differ from the tails of a normal distribution. The surface with a high kurtosis tends to have outliers or heavy tails. For the pressures (P) of 10, 30 and 40 MPa, kurtosis was similar to 3, for the remaining pressure of 20 MPa, the Sku parameter was higher; one can see that the ordinate distribution shown in Figure 3b is different from those obtained for other burnishing pressures. There is substantial difference between ordinate distributions of burnished surfaces and the probability distribution of the milled surface (Figure 3e). The values of skewness (0.4) and kurtosis (2.28) of the milled surface are typical for textures after cutting. In addition, the probability distribution of the milled surface is two-modal [35,36]. From the analysis of values of skewness and kurtosis and from the analysis of probability distributions of the analyzed textures (Figure 3), one can conclude that surfaces after burnishing are random, of the ordinate distributions similar to Gaussian, while the milled surface is deterministic.

The auto-correlation length (Sal) is the lowest for the surface after milling. From among burnished surfaces, the values of the Sal parameter were higher for rougher surfaces. This behavior is characteristic for random surfaces created by the same treatment. The texture–aspect ratio (Str) depends on surface isotropy. The values of the Str parameter of anisotropic surfaces were near 0, while the values of isotropic surfaces were near 1. The surface after milling was anisotropic (Figure 4e). The values of the Str parameter of burnished textures were between 40 and 83%. When the burnishing pressure was 20 MPa, the isotropic surface was machined, while for the other pressures, mixed surfaces were created. Rms. slope (Sdq) and arithmetical mean peak curvature were typically rougher for bigger surface heights.

An increase in surface topography height for burnishing pressures higher than 20 MPa was probably caused by significant plastic deformation of the disc surfaces.

## 4. Results of Tribological Tests and Discussion

**Figure 6:**
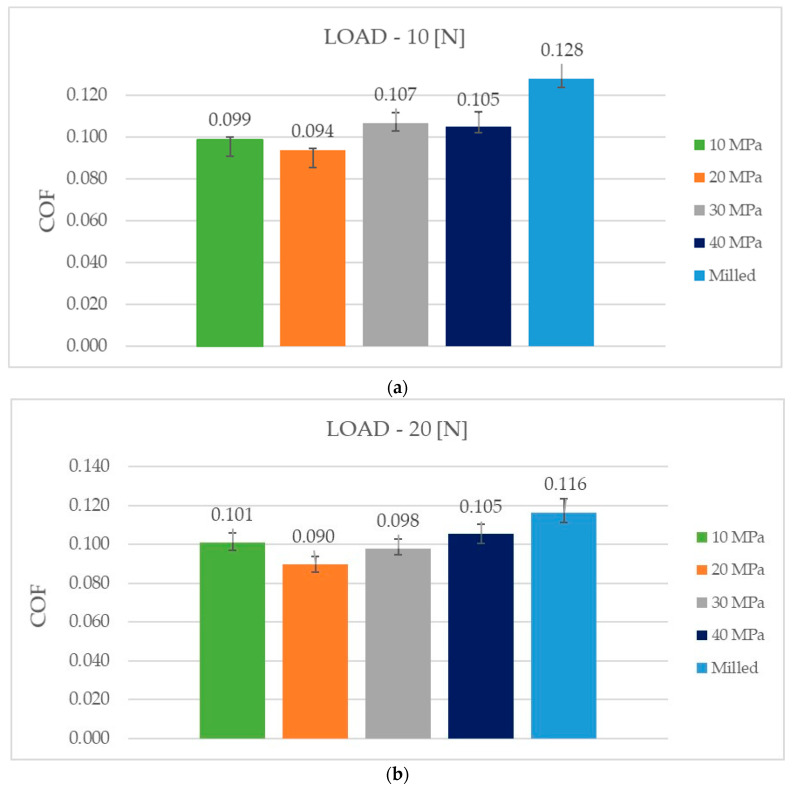

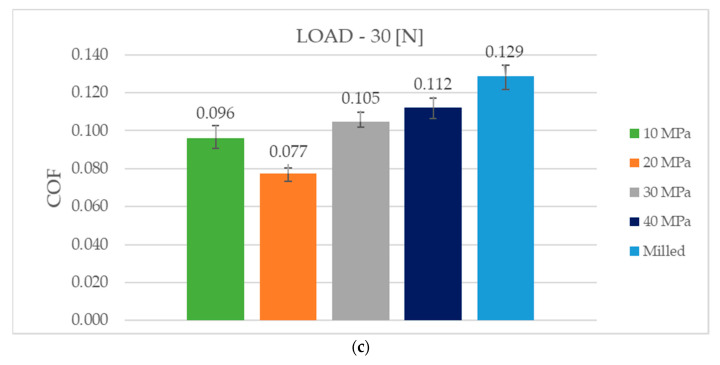
Average values of the friction coefficient for various assemblies under the load of 10 MPa (**a**), 20 MPa (**b**) and 30 MPa (**c**).

**Figure 7:**
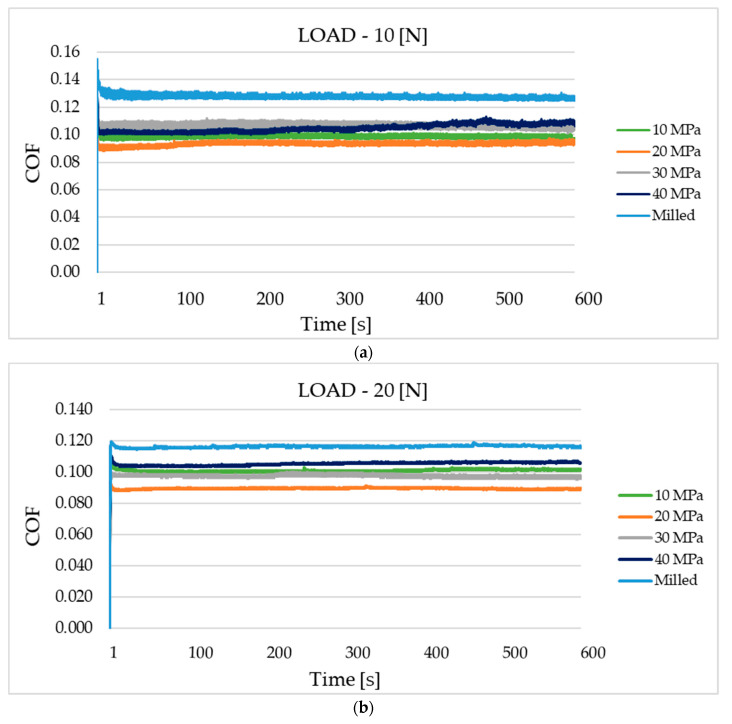

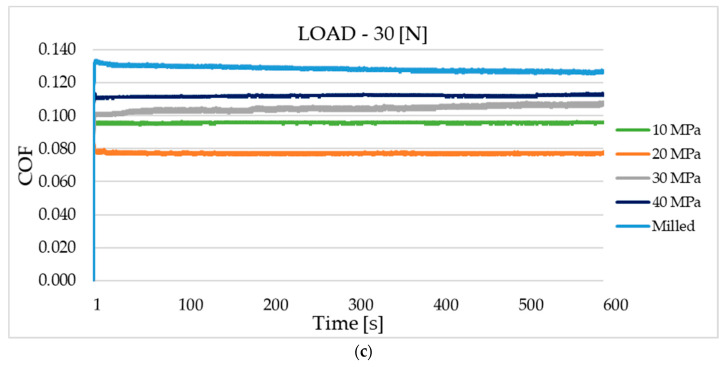
The friction coefficient against time for various sliding pairs under the load of 10 N (**a**), 20 N (**b**) and 30 N (**c**).

Average values of the coefficient of friction are presented in Figure 6. In order to exclude initial fluctuations, they were computed after five minutes of tests. Figure 7 presents examples of the coefficient of friction runs under different normal loads for various sliding assemblies.

Independently of the normal load applied, the burnishing treatment caused a decrease in the coefficient of friction in comparison to the sliding pair with the sample after milling. The largest decrease was achieved for assembly with the sample burnished under a pressure of 20 MPa. The values of the coefficient of friction were typical for mixed lubrication.

For the load of 10 N—Figure 6a, the highest reduction in friction, in comparison to assembly, which contained the disc after milling, was 26.6%. The application of the smallest burnishing pressure (10 MPa) led to smaller reduction in frictional resistance (of 22.6%). High burnishing pressures of 30 and 40 MPa led to comparatively high mean coefficients of friction (0.105–0.107), in these cases reductions of the resistance to motion were near 21%. The courses of the coefficient of friction versus time (Figure 7a) were different for sliding pairs with milled samples and for assemblies with burnished specimens. In the first case, after initial fluctuation, the friction force decreased to a small degree. When assemblies with burnished samples were tested, the coefficients of friction increased, as tests progressed. The final value of the coefficient of friction was the highest for the sliding pair with burnished discs with the highest pressure of 40 MPa.

When the medium normal load of 20 N was applied, the highest reduction in the coefficient of friction was 22.4%, due to the application of burnishing—Figure 6b. When pressures (P) were 10, 20 and 40 MPa, these reductions were similar to each other; they were a little higher than 10%. The worst results were received when the pressure (P) was the largest. After initial fluctuations, in most cases, the coefficients of friction obtained stable values. Some fluctuations were observed for assemblies with burnished discs with the pressures of 10 and 30 MPa (Figure 7b).

The highest reduction in the mean coefficient of friction due to burnishing was achieved for the highest normal load of 30 N—it was larger than 40% when the burnished pressure was 20 MPa. For assemblies with discs treated with the pressures of 10, 30 and 40 MPa, the decreases were 25.5, 18.6 and 13.2%, respectively—Figure 6c. Similar to the smallest load, the coefficient of friction of the sliding pair with the milled sample decreased with time. Different courses of the friction coefficient were observed when the burnished discs were tested. For burnishing pressures of 10, 20 and 40 MPa, after initial changes, the friction force was stable. However, when the burnished disc with the pressure of 30 MPa was tested, the coefficient of friction increased as the test progressed—Figure 7c.

The increase in the coefficient of friction with time of the burnished samples (Figure 7a,c) was probably caused by a reduction in the volume of oil in the contact zone. The milled sample with the biggest roughness height contained valleys, which retained lubricant. Therefore, in this case, the fiction force was stable or increased as the test progressed.

The obtained coefficient of friction was independent of the normal load used. The average coefficient of friction was near 0.1. This performance is probably related to low wear of the co-acting parts. Typically, due to better sliding surfaces matching, which resulted from an increase in the normal pressure, the coefficient of friction decreased for the higher normal load. Burnishing treatment led to the highest decrease in the coefficient of friction for the highest normal load of 30 N. Further research should be conducted with higher contact pressures.

**Figure 8:**
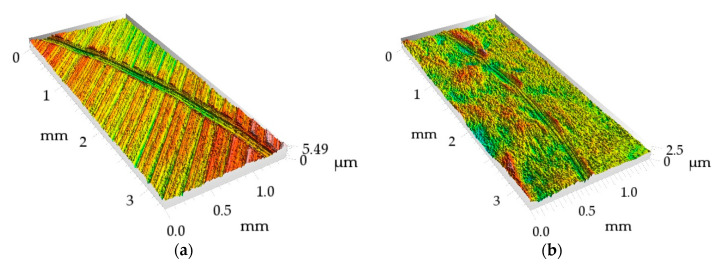
Isometric view of the milled surface (**a**) and of the burnished surface with a pressure of 20 MPa (**b**) after tribological tests at the normal load of 30 N.

**Figure 9:**
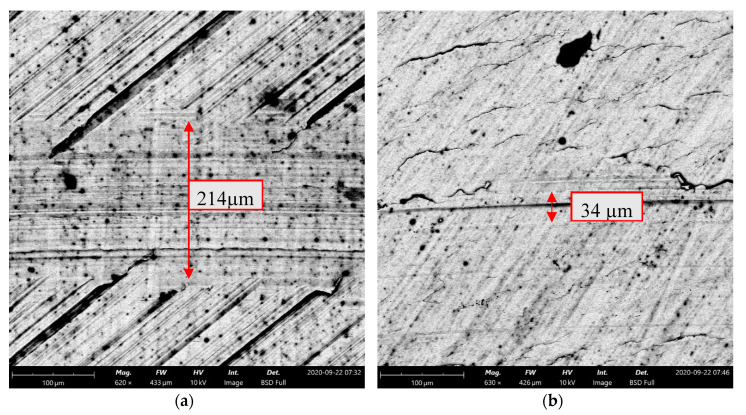
SEM views of the milled surface (**a**) and burnished surface with pressure of 20 MPa (**b**) after tribological tests at the normal load of 30 N.

As the tested sliding assemblies were lubricated and the test duration was low, it was difficult to measure wear levels of all tested discs. Wear tracks were more visible when normal loads were higher. It was found that when the coefficients of friction were higher, the sizes of the wear tracks were also larger. Figure 8 and Figure 9 present worn surfaces of the milled sample and the sample burnished with the pressure of 20 MPa. The milled sample led to the highest and the burnished sample led to the lowest value of the coefficient of friction (Figure 7c). One can see that both depth and width of wear track were higher for the milled sample compared to those of the burnished one—Figure 9. From the analysis of Figure 9, one can see that wear of disc had abrasive character.

The smallest friction coefficient of the assembly with the burnished disc with the pressure of 20 MPa was caused by the smallest height of surface texture. The Sq parameter of this sample was the smallest of all analyzed disc surfaces. It is also important that the surface slope (Sdq) was also the lowest. These parameters were about 40% smaller than those from the burnished surface with the pressure of 10 MPa. The average peak curvature (Spc) of the burnished surface with the pressure of 20 MPa is also much lower than that of the burnished surface with the lowest pressure. This means that the mean summit radius of the curvature of the burnished surface with the pressure of 20 MPa was more than two times larger. A common, contemporary friction model identifies two major sources of friction: the deformation of contacting asperities during relative motion and interfacial adhesion between the contacting asperities. In lubricated tests, the adhesion effect is negligible, while resistance to motion due to the deformation of contacting asperities is higher for the higher surface roughness of the contacting bodies. The very small rms. slope and large radius of asperity curvature of the burnished surface with the pressure of 20 MPa can also contribute to lower resistance to motion. Higher coefficients of friction of rougher samples in lubricated contact were also found in other works [20,21,22,23].

For the smallest and the largest normal forces, the coefficients of friction of assembly with the disc treated with the pressure of 10 MPa were smaller than those obtained for burnished discs with the pressures of 30 and 40 MPa. This tribological behavior was also probably caused by the smaller texture height, slope and the average asperity curvature of the disc treated with the pressure of 10 MPa, compared to those obtained for pressures of 30 and 40 MPa. For the medium and the highest normal load, the disc with the burnished surface with the pressure of 30 MPa led to smaller coefficient of friction compared to the disc surface treated with the highest pressure used—40 MPa. This behavior was also caused by the smaller roughness height of the disc burnished with the pressure of 30 MPa. The isotropic character of this surface (Str = 0.84) could be also the reason for the smaller resistance to motion of this surface compared to that of the surface treated with the highest pressure, this surface was characterized by smaller value of the Str parameter (0.56).

The ball burnishing process is applied to improve surface quality. An increase in the burnishing pressure from 10 to 20 MPa led to an improvement in surface roughness, and the amplitude parameters decreased. However, further increase in the burnishing pressure led to an increase in the surface topography height of the disc sample. This increase was probably caused by the plastic deformation, which caused the delamination of the surface layer.

Burnishing led to a decrease in the coefficient of friction of analyzed sliding pairs independently to the burnished pressure. The highest resistance to motion of the assembly with the milled sample was caused by the highest roughness height, characterized by the Sq parameter. The highest rms. slope (Sdq) and arithmetical mean peak curvature (Spc) of the milled surface can be also important. The anisotropic character of the milled sample can also cause high resistance to motion of the sliding assembly. A positive skewness of the milled sample can also influence high coefficients of friction, because this kind of surface contains more peaks than valleys. The beneficial effect of ball burnishing on the tribological behavior of sliding elements was also found in the previous investigation of the present authors under dry friction conditions [13].

## 5. Conclusions

In this paper, the effect of ball burnishing of steel disc samples on the coefficient of friction in lubricated sliding was studied. The authors of this paper have not found similar research studies in technical literature.

Ball burnishing of the surface after milling caused decreases in roughness height, slope and arithmetical peak curvature and led to increases in values of spatial parameters—Sal and Str. The smallest disc roughness height was achieved for the burnishing pressure of 20 MPa followed by the disc burnished with the pressures of 10, 30 and 40 MPa.

Ball burnishing in all analyzed cases caused a reduction in the friction coefficient, in comparison to that obtained for assembly with the sample after milling. Higher amplitude of texture corresponded to higher coefficient of friction. The best results were achieved for the sample burnished with the pressure of 20 MPa. Ball burnishing led to the highest decrease in the coefficient of friction (near 40%) for the highest normal load of 30 N.

It was impossible to assess the wear of all tested disc samples quantitatively. Wear tracks were more visible for larger normal loads. The larger coefficient of friction corresponded to higher wear levels of discs. Wear had abrasive characteristics.

## Figures and Tables

**Table 1:** Milling parameters.

Rotational Speed n, rev/min	Feed Per Tooth, vz, mm/Tooth	Depth of Cut Speed ap, mm	Feed Speed vf, mm/min
950	0.1	0.2	400

**Table 2:** Tribological parameters.

Normal Force, N	Sliding Speed, m/s	Test Duration, min
10, 20, 30	0.36	10

**Table 3:** Parameters of disc textures.

Disc	Burnished				Milled	Unit
Pressure	10 MPa	20 MPa	30 MPa	40 MPa		
Parameters	-	-	-	-		
Sq	0.25	0.16	0.57	0.81	0.87	µm
Ssk	−0.23	−0.21	0.12	0.12	0.41	-
Sku	3.01	4.28	2.92	3.2	2.28	-
Sp	0.79	1.02	2.11	2.75	2.66	µm
Sv	1.11	0.83	1.97	3.06	2.03	µm
Sal	0.19	0.09	0.29	0.38	0.05	mm
Str	0.4	0.56	0.84	0.56	0.03	-
Sdq	0.024	0.014	0.059	0.052	0.072	-
Spd	392	122	770	280	462	1/mm^2^
Spc	14.03	5.89	51.4	49.4	53.9	1/mm
